# Treatment of extrahepatic biliary fistulas using n-butyl cyanoacrylate

**DOI:** 10.1590/0100-3984.2018.0004

**Published:** 2019

**Authors:** Thiago Franchi Nunes, Tiago Kojun Tibana, Márcio Eduardo de Souza Pereira, Edson Marchiori

**Affiliations:** 1 Universidade Federal de Mato Grosso do Sul (UFMS), Campo Grande, MS, Brazil.; 2 Hospital Regional de Mato Grosso do Sul, Campo Grande, MS, Brazil.; 3 Universidade Federal do Rio de Janeiro (UFRJ), Rio de Janeiro, RJ, Brazil.

## INTRODUCTION

Biliary fistula is a serious complication that requires rigorous evaluation for objective and safe determination of the therapeutic procedure of choice. The main causes are surgical procedures and trauma, which account for 67% and 19% of cases, respectively^(^^1^^)^. Traditionally, biliary fistulas are treated surgically. Recent advances in interventional radiology have provided a safe alternative to surgical treatment for lesions of the biliary tract, making it possible to perform procedures that are highly efficacious and less invasive^(^^2^^-^^5^^)^.

Reoperation is often difficult, mainly due to adhesions. Another relevant factor is the anesthesia procedure for patients with biliary fistula, who are often clinically unstable. Therefore, many authors have stated that there is a need for an alternative therapeutic approach^(^^6^^,^^7^^)^. Percutaneous transhepatic cholangiography, guided by fluoroscopy and performed under conscious sedation and local anesthesia, might be a low-risk option to avoid unnecessary surgery, as well as being better tolerated by most patients^(^^3^^)^. In the last decade, various interventional radiology techniques for the treatment of biliary fistulas, such as embolization with liquid agents, have been described^(^^8^^,^^9^^)^. Injection of n-butyl cyanoacrylate surgical glue is a safe procedure that produces and excellent results , especially in patients with isolated segmental bile duct complications^(^^8^^)^.

Biliary fistulas are often accompanied by non-dilated extrahepatic bile ducts, and the procedure can therefore be technically difficult. Consequently, it is preferable that they be evaluated and treated by experienced interventional radiologists^(^^10^^)^.

## PROCEDURE

In general, the biliary tract is not dilated in cases of extrahepatic fistula, making the treatment more technically difficult^(^^10^^)^.

**Figure 1:**
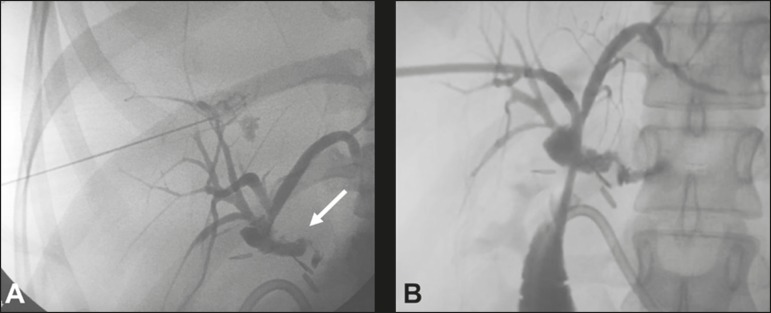
**A:** Puncture of the right bile duct with a 22-gauge Chiba needle and cholangiography demonstrating a break in the continuity originating in the hepatobiliary duct, near the biliary-enteric anastomosis, corresponding to an extrahepatic biliary fistula (arrow). **B:** Placement of a 12-F biliary drain with its end positioned in the jejunal loop. Note the persistence of the fistulous tract.

Initially, the bile duct is punctured with a 22-gauge Chiba needle, by the classically described technique^(^^8^^)^, on the basis of previous imaging tests (Figure 1A). Cholangiography is performed in order to visualize the fistulous tract. That is followed by the insertion of a biliary drain (pigtail catheter, 12-F or larger) (Figure 1B), with the objective of complete occlusion of the hepatobiliary duct, in order to avoid extravasation of the surgical glue into the intrahepatic biliary tract, the end of the drain being positioned in the jejunal loop.

**Figure 2:**
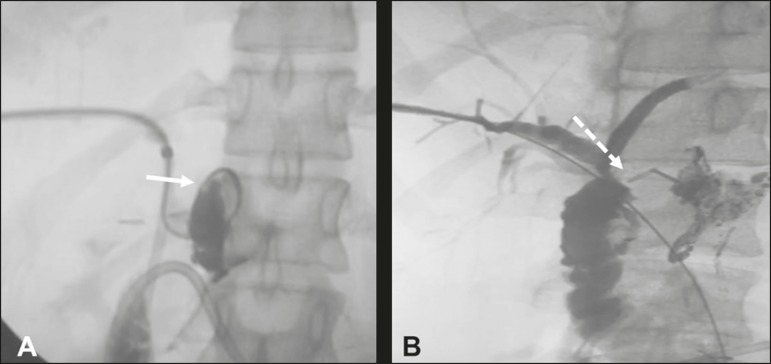
**A:** Catheterization of the fistula with a 2.9-F microcatheter and embolization with surgical glue (arrow). **B:** Follow-up cholangiography obtained 30 days after the procedure, showing adequate emptying of the biliary tract and complete closure of the fistulous tract (arrow).

Using the orifices closest to the drainage holes, the fistula is catheterized with a 2.9-F microcatheter, the correct positioning of which is confirmed by infusion of contrast medium. The microcatheter is then irrigated with 5% dextrose prior to the procedure, preventing the surgical glue from agglutinating therein. Embolization is performed with a solution of 2 mL of n-butyl cyanoacrylate diluted in 10 mL of lipiodol (1:5) (Figure 2A). In this specific case, we opted for greater dilution, aiming to occlude the hepatobiliary duct to the most distal point possible, thus favoring the administration of a larger volume of the cyanoacrylate/lipiodol solution and reducing risk of its migration into the biliary tract. After embolization, 5 mL of dextrose are infused via the microcatheter, subsequently being slowly and carefully extracted via the biliary drain. At the end of 30 days, a follow-up cholangiogram is obtained in order to confirm the closure of the fistula (Figure 2B). If closure is confirmed, the biliary drain is removed.

## References

[1] Jablonska B, Lampe P (2009). Iatrogenic bile duct injuries: etiology, diagnosis and
management. World J Gastroenterol.

[2] Nasser F, Rocha RD, Falsarella PM (2016). Percutaneous treatment of intrahepatic biliary leak: a modified
occlusion balloon technique. Cardiovasc Intervent Radiol.

[3] Righi D, Franchello A, Ricchiuti A (2008). Safety and efficacy of the percutaneous treatment of bile leaks
in hepaticojejunostomy or split-liver transplantation without dilatation of
the biliary tree. Liver Transpl.

[4] Zurstrassen CE, Bitencourt AGV, Guimaraes MD (2017). Percutaneous stent placement for the treatment of malignant
biliary obstruction: nitinol versus elgiloy stents. Radiol Bras.

[5] Cardarelli-Leite L, Fornazari VAV, Peres RR (2017). The value of percutaneous transhepatic treatment of biliary
strictures following pediatric liver transplantation. Radiol Bras.

[6] Ng S, Tan KA, Anil G (2015). The role of interventional radiology in complications associated
with liver transplantation. Clin Radiol.

[7] Civelli EM, Meroni R, Cozzi G (2004). The role of interventional radiology in biliary complications
after orthotopic liver transplantation: a single-center
experience. Eur Radiol.

[8] Vu DN, Strub WM, Nguyen PM (2006). Biliary duct ablation with N-butyl cyanoacrylate. J Vasc Interv Radiol.

[9] Park JH, Oh JH, Yoon Y (2005). Acetic acid sclerotherapy for treatment of a biliary leak from an
isolated bile duct after hepatic surgery. J Vasc Interv Radiol.

[10] Kühn JP, Busemann A, Lerch MM (2010). Percutaneous biliary drainage in patients with nondilated
intrahepatic bile ducts compared with patients with dilated intrahepatic
bile ducts. AJR Am J Roentgenol.

